# Can clinician champions reduce potentially inappropriate medications in people living with dementia? Study protocol for a cluster randomized trial

**DOI:** 10.1186/s13012-022-01237-0

**Published:** 2022-09-27

**Authors:** Michael L. Parchman, Jennifer Perloff, Grant Ritter

**Affiliations:** 1 Kaiser Permanente Washington Health Research Institute, Seattle, WA USA; 2 Brandeis University, Waltham, MA USA; 3 Institute for Accountable Care, Washington, DC USA

**Keywords:** De-implementation, De-prescribing, Low-value, Overuse, Dementia, Implementation science

## Abstract

**Background:**

For people living with dementia (PLWD) the overuse of potentially inappropriate medications (PIMs) remains a persistent problem. De-prescribing trials in the elderly have mixed results. Clinician champions may be uniquely suited to lead efforts to address this challenge. Here we describe the study protocol for a 24-month embedded pragmatic cluster-randomized clinical trial within two accountable care organizations (ACOs) of such a clinician champion intervention. The specific aims are to (1) assess the effectiveness of a clinician champion on de-implementing PIMs in PLWD, (2) determine if the intervention is associated with a reduction in emergency department (ED) visits and hospitalizations attributed to a fall, and (3) examine five implementation outcomes: appropriateness, feasibility, fidelity, penetration, and equity.

**Methods/design:**

Two ACOs agreed to participate: United States Medical Management (USMM) and Oschner Health System. The unit of randomization will be the primary care clinic. A clinician champion will be recruited from each of the intervention clinics to participate in a 6-month training program and then work with clinicians and staff in their clinic for 12 months to reduce the use of PIMs in their PLWD population. For aims 1 and 2, Medicare claims data will be used to assess outcomes. The outcome for aim #1 will be medication possession rates per quarter, for the three therapeutic classes of PIMs among patients with dementia in intervention clinics versus control clinics. For aim #2, we will assess the incidence of falls using a previously validated algorithm. For both aims 1 and 2, we will construct hierarchical models with time period observations nested within patient using generalized estimating equations (GEE) with robust standard errors. The key variable of interest will be the treatment indicator assigned based on practice. For aim #3, we will conduct qualitative thematic analysis of documentation by the clinician champions in their project workbooks to evaluate the five implementation outcomes.

**Discussion:**

This embedded pragmatic trial will add to our existing knowledge regarding the effectiveness of a clinician champion strategy to de-prescribe potentially inappropriate medication among patients with dementia as well as its appropriateness, feasibility, fidelity, penetration, and equity.

**Trial registration:**

Clinicaltrials.govNCT05359679, Registered May 4, 2022

Contributions to the literature

- Research on de-implementation strategies to support de-prescribing in the elderly have mixed results. This is concerning given the persistently prevalent use of potentially inappropriate medications (PIMs) among people living with dementia.
- Clinician champions are often used to support implementing evidence into practice, but much less is known about their effectiveness in de-implementing a low-value care service such as PIMs for people living with dementia.
- This pragmatic embedded cluster-randomized trial conducted within two accountable care organizations will assess the effectiveness of a clinician champion in decreasing the use of PIMs by prescribers in their clinic for people living with dementia and evaluate appropriateness, feasibility, fidelity, penetration, and equity.

## Background

For people living with dementia (PLWD), the overuse of potentially inappropriate medications (PIMs), those for which the potential for harm outweighs benefit, remains a persistent problem despite evidence-based guidelines supporting their de-adoption [1–3]. These harms can include falls, cognitive impairment, hospital admission, functional impairment, and death. A group of geriatric experts convened by the Choosing Wisely initiative identified three classes of PIMs for PLWD: antipsychotics, benzodiazepines, and hypoglycemics (sulfonylureas and insulin) with adequate glycemic control [3, 4]. Here, we describe the study protocol for a 24-month embedded pragmatic cluster-randomized clinical trial within two large accountable care organizations (ACOs) of a clinician champion intervention to decrease the use of PIMs in PLWD funded by the National Institute of Aging’s IMbedded Pragmatic Alzheimer’s disease (AD) and AD-Related Dementias (AD/ADRD) Clinical Trials (IMPACT) Collaboratory [5].

The reported prevalence of any PIM when cognitive impairment is reported ranges from 20.6 to 80.5% [3]. Approximately 14.3% of Medicare Part D enrollees with dementia residing in the general community are prescribed an antipsychotic [6]. The prevalence of potentially inappropriate benzodiazepine prescriptions has been reported to be as high as 20% among elderly persons with dementia living in the community [7]. Another study found that the proportion of elderly patients with an A1c < 7% who received a prescription for sulfonylurea, insulin or combined insulin and sulfonylurea therapies was 35.2%, 24.2%, and 16.3% respectively and was as prevalent in those with dementia as in those without [8]. A study of comparing rates of PIM prescribing in traditional Medicare and Medicare Advantage populations from 2006-2015 found no difference in rates between the two groups [9]. In addition, there was no evidence of any decline in rates of prescribing over time. Given these findings, what can a health system do to de-implement PIMs among their PLWD population?

De-prescribing trials, either with the elderly or specific to patients with dementia, have mixed results [10–16]. The EMPOWER trial demonstrated that pharmacist-led shared decision-making conversations led to a significant reduction in inappropriate benzodiazepine use among community-dwelling older adults [17]. In the D-PRESCRIBE trial, a pharmacist led educational outreach intervention to both patients and their primary care clinician resulted in a significant decrease in the use of potentially inappropriate medications in a community-dwelling population of older adults [18]. However, the recently published OPTIMIZE trial reported no significant changes in numbers of long-term medications or number of PIMs after a light-touch educational de-prescribing intervention for older adults with cognitive impairment taking 5 or more long-term medications and their primary care clinicians [19].

Developing and supporting a clinician champion is a simple and pragmatic implementation strategy that might be effective in addressing the overuse of PIMs in the PLWD population. Factors affecting de-implementation are multi-level, complex, context specific, and interact in ways that are different than implementation of an evidence-based service [20]. There is a clear rationale for why clinician champions are uniquely suited to lead a multi-level context-specific de-implementation change effort [21]. They are front-line clinicians who can advocate for and influence practice-driven change and may be particularly effective in de-implementing PIMs because they can (1) be a trusted source of information for colleagues and patients about the potential harms of PIMs, (2) provide feedback and discuss prescribing behaviors with their colleagues, (3) serve as a role model for how to provide guideline-concordant care for PLWD [22], and (4) identify practice-specific barriers and facilitators and leverage them to reduce PIMs use in their local setting [23]. Use of a clinician champion is widely recognized as effective in implementing evidence-based practices in health care settings [21]. Less is known about their effectiveness in de-implementing a low-value service such as PIMs among PLWD [24].

With support from the Robert Wood Johnson Foundation, (RWJF) six clinician champions from safety net care settings across the USA recently completed a fellowship training program to provide them with the core competencies they needed to be effective in reducing an overused service in their setting. A group of national experts with experience in de-implementing low-value care served as faculty. Work by these value champions resulted in declines in the use of triple lumen peripherally inserted central venous lines in the inpatient setting, imaging for low back pain in the emergency department, prescribed cough and cold medicines for infants and children in a primary care setting and prescribing long-term opioids for patients with chronic pain. The six most important implementation strategies used by the champions to support de-implementation were building a coalition of partners in the effort, conducting a local needs assessment, developing a formal implementation blueprint, conducting educational meetings, facilitating conversations about the overused service, and implementing clinical reminders [25]. A formative and summative evaluation of the Fellowship resulted in the development of a training curriculum and a project workbook to guide the work of a clinician champion during a low-value care project.

For this study clinician champions will participate in training using the RWJF fellowship curriculum and lead an initiative within their primary care clinic setting to decrease the use of the three classes of PIMs described above for PLWD: antipsychotics, benzodiazepines and hypoglycemic medications. The specific aims are to (1) assess the effectiveness of a clinician champion on de-implementing these three classes of PIMs, (2) determine if the intervention is associated with a reduction in emergency department (ED) visits and hospitalizations attributed to a fall, and (3) examine five implementation outcomes critical to the success of the de-implementation effort: appropriateness, feasibility, fidelity, penetration, and equity.

## Methods

### Settings, subjects, and recruitment

Accountable Care Organizations (ACOs) are designed to promote patient-centered care and reduce the use of unnecessary services, making them a natural setting to test the effectiveness of a clinician value champion intervention. Co-investigators with the Institute for Accountable Care (IAC), which is the research arm of the National Association of Accountable Care Organizations, (NAACOS), worked with NAACOS to recruit two ACOs for this study: United States Medical Management (USMM) and Oschner Health System. Clinicians with the Visiting Physicians Association contract with USMM to conduct home-based primary care from 39 offices across 13 states. Teams in each office consists of a provider/medical assistant dyad who is also supported by a patient care coordinator, a scheduler, and a nurse navigator who does outreach to more complex patients. Within each office, a medical team meets weekly to discuss patient cases and update their approach to patient care. Over 30% of the Medicare population they served in 2019 (approximately 6000 patients) had an established diagnosis of dementia (Table 1). Oschner Health System is in Louisiana and has 28 primary care clinic locations within their integrated group practice. Their Medicare ACO population had 31,525 enrollees in 2019 and within this population 1387 (4.4%) had a documented diagnosis of dementia (Table 1).

**Table 1:** Medicare beneficiaries with dementia in each accountable care organization

	Ochsner Health System		USMM^a^	
	Number	Percent	Number	Percent
Unique Beneficiaries, 2019	31,525	–	19,493	–
Unique Beneficiaries with Dementia	1387	4.40%	6639	34.06%
Unique Beneficiaries with Incident Dementia	353	1.12%	1015	5.21%
Unique Beneficiaries with Prevalent Dementia	1752	5.56%	7653	39.26%
Unique Beneficiaries with Part D Eligibility	9831	31.18%	11,154	57.22%

^a^United States Medical Management

### Randomization

A schedule of enrollment, randomization and allocation, interventions, and assessments for the study is found in Table 2. The unit of randomization will be the primary care clinic, and outcomes for aims 1 and 2 will be assessed by comparing patients with a diagnosis of dementia who receive care in clinics assigned to the intervention arm to those who receive care in clinics assigned to the control arm of the study. We anticipate 32 out of 39 teams from USMM to meet eligibility and 26 out of 28 practices for Ochsner Health. Within each ACO, we will randomly select half of the practices for the intervention and the remaining practices will continue with usual care as controls. Prior to random assignment, we will stratify practices based on size using data for 2019–2020. Practices will be paired by number of unique beneficiaries treated (first and second largest practice, third and fourth largest, and so on) and then one member of each pair will be randomly selected via a random number generator to be a treatment site. Once randomized, a clinician champion will be recruited from each of the offices or clinics in the intervention arm of the study in partnership with the ACO leadership to participate in the clinician champion training program. Neither investigators nor participants will be blinded to allocation. The random allocation sequence and assignment to study arms will be done by the statistician (GR) at Brandeis University.

**Table 2:** Schedule of enrollment, interventions, and assessments

	2021		2022				2023	
	July–September	October–December	January–March	April–June	July–September	October–December	January–March	April–June
**Enrollment**								
Identify and randomize clinics	X							
Recruit clinician champions	X	X						
**Intervention**								
Clinician training webinars			X	X				
Clinician champions work to reduce PIMs in PLWD				X	X	X	X	
Monthly shared learning calls Post-training intervention					X	X	X	
**Assessments**								
Medicare claims data analysis (aims 1 and 2)							X	X
Collect clinician champion project workbooks and analysis (aim 3)							X	X
**Dissemination**								X

### Intervention

Each clinician champion will participate in one-hour training webinars over 6 months (January–June 2022) using the curriculum developed in the afore-mentioned RWJF Value Champions fellowship program. These sessions will be led by the PI (MP) who also was the Director of the RWJF value champion fellowship from which these materials were developed. The Learning Modules (Table 3) follow the same format and include a rationale, quotes from RWJF value champion fellows, learning objectives, required readings, discussion questions, application of the learnings to their project workbook, and additional resources. Each Learning Module is also accompanied by a Facilitator/Instructors Guide so that any health care organization can use these materials to train their own value champions. Near the end of the training webinars, each value champion will launch his/her own 12-month PIM de-prescribing project (April 2022–March 2023) using the Project Workbook as their guide. Following the 6 months of training clinician champions will participate in a monthly 1-h shared learning webinar to share successes, challenges, and brainstorm solutions during the 12 months when champions are doing the work to support reductions in the use of PIMs in PLWD.

**Table 3:** Value champion learning modules

**Module 1: High value care and health equity:** Understanding the intersection between “value” and “equity” can inform conversations between value champions, leadership and stakeholders and inform project planning.	
**Module 2: Engaging leadership for high value care:** Provides participants with an overview of how to engage leadership throughout the project and provides practical tips on how to create and sustain this engagement.	
**Module 3: Choosing an overused service:** Overuse of low-value care is common, selecting a target area of overuse requires careful consideration of multiple factors that will impact subsequent success.	
**Module 4: How to conduct a stakeholder assessment and why it is important:** Identifying, assessing and engaging stakeholders who will be impacted by reducing the use of a service is critical to project success.	
**Module 5: Engaging patient participation in your project:** This module explains why incorporating the patient voice is important in reducing overuse and provides examples of how to do so.	
**Module 6: Measurement, data and trust, supporting change with data:** This module discusses common challenges and a range of solutions for collecting and reporting overuse data when taking action on overuse.	
**Module 7: Engaging health care professionals:** The ‘Taking Action on Overuse’ framework and change package provides suggested key activities to select from when designing an intervention.	
**Module 8: Strategies employed by value champions:** This module provides examples of intervention strategies used by clinician value champions in their setting to engage providers, staff, and patients in change efforts.	
**Module 9**: **Choice architecture and overuse reduction:** This module helps participants understand how concepts from the field of behavioral economics can be applied to their overuse reduction initiative.	
**Module 10**: **Planning for sustainment:** An understanding of how value champions can incorporate plans for sustainment into every aspect of their overuse reduction initiative is provided in this module.	

### Data sources and collection

For aims 1 and 2, de-identified Medicare claims data will be used to assess outcomes. Co-investigators from the Institute for Accountable Care (IAC) will access Medicare claims data in the CMS Virtual Research Data Center (VRDC) repository and claims provided to ACOs (Claim and Claim Line Feed File or CCLF files). Specifically, we will use quarterly Medicare clinician bills (Part B), institutional bills (Part A), and prescription drug event (PDE) data (Part D) for diagnostic and utilization information on Medicare beneficiaries and practices for 2019 and 2020. Data for 2021 and 2022, including prescription drug events data, clinician bills and institutional bills will be derived from the ACO’s CCLF files which are provided by CMS monthly. For aim #3, the five implementation outcomes will be assessed by using available project management data recorded by each value champion in their project workbook, specifically the milestones completed within and across each phase of work outlined in the workbook.

Following an intent-to-treat design, all Medicare beneficiaries meeting our dementia eligibility criteria will be included in the study, regardless of level of engagement with practice clinicians. Specific criteria include the following:

1. Assigned to one of the participating ACO during the study period;
2. Continuously enrolled in Medicare Parts A, B, and D;
3. Have dementia as defined in Medicare claims data using the Bynum-Standard algorithm [26]; and
4. Have one or more evaluation and management visit at a participating practice during the study period.

Medicare beneficiaries will be excluded if they have any months of Part C (Medicare Advantage) during study period since prevents us from seeing all of their care. All data will be available to all study investigators.

### Data analysis

#### Specific aim #1 analysis

The primary outcome for this study will be medication possession rates (MPR) per quarter, for the three therapeutic classes of PIMs. These beneficiary-level measures are calculated as quotients with denominator equal to the length of the quarter and the numerators equal to the days supplied for prescriptions within the class filled during the quarter, plus excess days-supply from the previous period minus excess days-supply remaining at the end [27]. Overlapping prescriptions from within the same therapeutic class will be only counted once. Unlike many studies using medication possession rates to exam adherence, we are interested in reducing, not increasing, the percent of days covered. We will initially try to model the originally valued MPR variables, but may have to transform the scores to ordinal variables based on the ‘degree’ of deprescribing. For example, Benner and colleagues in their study of statin adherence defined non-adherence as MPRs within 0–20%, low adherence as between 21 and 79% and high adherence as 80% or higher [28]. In addition to MPRs, we will also calculate and model the number of 30-day prescriptions per time period for each beneficiary in the study.

Data analysis will begin with descriptive statistics, comparing the mix of patients at each practice including demographics, chronic conditions, and incident versus prevalent dementia. This will allow us to check for balance between treatment and control practices and inform subsequent modeling. To determine the impact of the intervention, we will construct hierarchical models with time period observations nested within patient. These models will be random effects or generalized estimating equations (GEE) with robust standard errors, and will be either linear, if original MPRs are used as outcome, or generalized linear, if transformation necessary. The key variable of interest in these models will be the treatment indicator (assigned based on practice). Since there could well be a time effect of treatment, we will explore several model specifications, such as discrete indicators for the time periods or a single continuous variable. Interactions of treatment with time period will also be explored. By examining various different specifications involving time, we should be able to determine whether the intervention leads to a one-time change, a gradual trend over time, or a combination of both (an immediate jump with a further change, as time passes). Finally, we will explore the impact of this intervention on health inequities by stratifying our primary analysis by race/ethnicity using indicators from the Medicare beneficiary summary file (MBSF).

As a secondary outcome (aim #2), we will assess the incidence of falls during the study window using an algorithm developed by Min and colleagues [29]. Specifically, the measure looks for direct indicators of fall (e.g., ‘reason for visit’ codes) or the presence of injuries most likely caused by a fall (spiral fracture to the radius) on all provider (part B) and institutional (part A) claims. Key covariates in all of our regression models will include age and sex, and reason for entitlement since these may be out of balance given the small number of practices being randomized. We will also include a fixed effect for ACO.

For specific aim #3, we will have a total of 30 clinician value champions across the 2 ACOs who will share their completed project workbooks to assess the five implementation outcomes. Current approaches to evaluating implementation outcomes are still nascent, with few measures that have validated psychometric properties [30, 31]. We will report each outcome, for example, the number of feasibility milestones completed, as an overall proportion or their central tendency such as a mean value with standard deviation, both within each ACO and across the two ACOs. Conceptually, these measures are inter-dependent, and are connected with service outcomes, such as patient safety, measured here by exposure to a PIM within our study population of PLWD [32]. In addition, notes and recordings from monthly shared learning calls will be used to conduct a thematic analysis using a deductive approach using the five outcomes described in Table 4 [33].

**Table 4:** Implementation outcomes

Definitions	Source of data	Measure
Appropriateness: *“…”appropriateness” is deemed important for its potential to capture some “pushback” to implementation* efforts.”	Project Workbook: stakeholder power grid completed in “Understand Project Landscape?	Stakeholder Analysis Power Grid: proportion of stakeholders who are opponents of the project or express reservations
Fidelity: *“…is the degree to which an intervention was implemented as it was prescribed in the original protocol or as it was intended by the program developers”*	Project Workbook: completed milestone log	Total number of milestones met across all 7 phases of work; how many phases of work with at least 50% of milestones completed
Feasibility: “…*the extent to which a new innovation, can be successfully used or carried out within a given agency or setting.”*	Project Workbook: Milestones log	Number of milestones completed in “Test the Intervention” phase of work
Penetration: *“…the integration of a practice within a service setting and its subsystems.”*	Project Workbook: stakeholder analysis worksheet log	Number of champions who complete the intervention. Number of stakeholders identified and engaged by each champion.
Equity: “…*proactive tailoring of de-implementation strategies to address healthcare inequities and unintended consequences for vulnerable populations*.”	Project Workbook: Milestones log.	Three milestones have an equity focus, one each within phases 2, 5, and 7. Number of equity milestones completed in Workbook Phases 2, 5, and 7.

Workbook data will also be used to understand their activities as documented in their responses to journal question prompts and assess their fidelity to the intervention by examining the proportion of milestones reached for each phase of their work as documented in the workbook [33]. If the workbook data and notes and recordings from monthly shared learning calls prove to be inadequate, we will conduct structured interviews with a sub-set of clinician champions near the end of the intervention period to identify their activities, assess fidelity, and evaluate implementation outcomes.

### Sample size and power

Our sampling plan anticipates enrolling at least 30 of the 39 practices in USMM and 26 of the 28 practices in Ochsner. Based on Medicare enrollments of 39,000 members in USMM with a 20% dementia rate in 2019, and 10,000 members in Ochsner with approximately 10% of members diagnosed with dementia om 2019, we should have at least 2000 patients per treatment group for USMM and 500 patients per treatment group for Ochsner. For our primary outcome, with a follow-up period of 1 year and with mediation possession rates calculated per patient each quarter, we will have approximately 4 MPR observations per patient or 8000 observations per group for USMM analyses and 2000 per group for Ochsner. Even after assuming significant correlation across time within patient and modest correlation between patients within a practice, which will reduce effective sample sizes by 20%, we should have the equivalent of 6400 observations per treatment group for our USMM analyses and 1600 observations per treatment group for Ochsner. The expectation of our intervention is that for each medication class, it will produce clinically relevant decreases in mean possession rates of 10% of a standard deviation. If the effect size is this large for a medication class and using a 5% level of significance, our Ochsner sample (with effective size of at least 1600 per group) will have 80% power to discern a significant difference, while our larger USMM sample (with effective size of at least 6400 per group) will have almost 100% power. Indeed, our USMM sample will have 80% power to find a significant difference, even if the effect size is only 5% of a standard deviation. Ethics approval for this study was obtained through the IMPACT Collaboratory’s central institutional review board, Advarra, on September 8, 2021.

### Data monitoring

The NIA IMPACT Collaboratory’s Data Safety Monitoring Board will provide oversight and review. The data safety monitoring plan includes monthly adverse event reporting by each clinician champion for patients with dementia seen in clinics randomized to the intervention arm. All adverse events that are serious and unexpected will be reported to the IMPACT Collaboratory Regulatory and Data Team Leader, NIA IMPACT Collaboratory Project Officer, and the project’s Safety Officer within 48 h of the study’s knowledge of SAE. Since adverse event reporting is not available for patients in the control arm, we will provide the board with quarterly reports comparing all emergency department visits, inpatient admissions and mortality among patients with dementia who are seen in clinics randomized to the intervention arm of the study and those seen in clinics randomized to the control arm of the study.

### Ethics approval

The Institutional Review Board (IRB) for the NIA IMPACT Collaboratory, Advarra, reviewed and approved the study protocol on September 8, 2021. A waiver of consent was approved for use of Medicare claims data to conduct the analyses for aims 1 and 2 was approved. Clinician champions were provided a study information sheet at the start of the intervention (January 2022) with information about their participation and how to withdraw from the study. Any subsequent protocol modifications will be submitted to the Advarra IRB.

## Discussion

### Significance

In their commentary on unpacking de-implementation, Norton and Chambers encouraged the field of implementation science to consider “critically important distinctions between the implementation of new, evidence-based interventions, and the de-implementation of currently delivered inappropriate interventions.” [20]. Although there is a strong rationale for the use of a clinician champion as a de-implementation strategy [24], few studies have employed them to address low-value care, although Colla and colleagues identified strategies that champions might employ such as clinician education and provider feedback as effective [34]. Santos and colleagues recently completed a systematic review of the effectiveness of a clinician champion when implementing an innovation into health care settings [35]. Only one of the 35 studies included in the review was focused on de-implementing a low-value care service, that of urinary catheters in pediatric patients. However, it is possible that the search strategy focus on implementation for this systematic review did not identify studies of de-implementation. This embedded pragmatic trial will add to the literature and expand our understanding of the effectiveness of a clinician champion as a strategy to de-implement care for which the potential for harm is greater than the potential for benefit: potentially inappropriate medications prescribed to people living with dementia.

This study will also allow health care systems and investigators to gain real-world experience in developing clinicians who can be champions of high-value care within their setting. Leaders in both participating ACOs have expressed an interest in spreading this intervention to all primary care clinic locations within their ACO and to other members of National Association of Accountable Care Organizations. Both findings from the trial and lessons learned from preparing clinician champions will be disseminated through publications in peer-reviewed publications, presentations at national conferences, and for interested stakeholders such as ACO leadership.

### Limitations

It is possible that clinician champions may leave or relocate their clinical practice during the study. We will guard against this be identifying a primary and secondary clinician champion within each clinic. Information shared by the clinician champions within their own clinical setting in the intervention arm of the study may be inadvertently shared with clinicians who provide care in clinics randomized to the control arm of the study. Finally, there may be clinics with a small sample of patients with dementia, which may adversely impact our ability to draw conclusions from the results.

### Impact

Value champions who complete the study will be prepared to serve as mentors of clinician value champions within their own organizations and across other ACOs after the conclusion of the study. The clinician champion training is generic and can be applied to any low-value care service and the content of the curriculum, along with the project workbook, have been deliberately designed so that any health system might use them to train their own cohort of clinician champions in the future.

## Data Availability

The curriculum and materials to prepare clinician champions for the intervention are available at https://www.act-center.org/our-work/primary-care-transformation/high-value-care/taking-action-on-overuse/value-champions-training-curriculum. The datasets will be available upon request from the investigators.
